# Early Nutritional Intervention in Patients with Non-Small Cell Lung Cancer Receiving Concurrent Chemoradiotherapy: A Phase II Prospective Study

**DOI:** 10.3390/nu17081389

**Published:** 2025-04-21

**Authors:** Fangjie Liu, Qiaoting Luo, Yu Xi, Pengxin Zhang, Yingjia Wu, Suping Guo, Yaoling Dong, Daquan Wang, Qingping Wu, Hui Liu, Yuming Rong, Bo Qiu

**Affiliations:** 1 Department of Radiation Oncology, State Key Laboratory of Oncology in South China, Guangdong Provincial Clinical Research Center for Cancer, Sun Yat-sen University Cancer Center, Guangzhou 510060, China; liufj@sysucc.org.cn (F.L.);; 2 Guangdong Association Study of Thoracic Oncology, Guangzhou 510000, China; 3 Guangdong Provincial Key Laboratory of Microbial Safety and Health, State Key Laboratory of Applied Microbiology Southern China, Institute of Microbiology, Guangdong Academy of Sciences, Guangzhou 510070, China; 4 Department of Traditional Chinese Medicine, State Key Laboratory of Oncology in South China, Guangdong Provincial Clinical Research Center for Cancer, Sun Yat-sen University Cancer Center, Guangzhou 510060, China

**Keywords:** locally advanced non-small cell lung cancer, concurrent chemoradiotherapy, nutritional intervention, gut microbiota, survival outcome

## Abstract

**Aims:** This phase II study aimed to evaluate the impact of early nutritional intervention on the nutritional status and survival of locally advanced non-small cell lung cancer (LANSCLC) patients undergoing concurrent chemoradiotherapy (CCRT). **Methods**: LANSCLC patients treated with CCRT were enrolled in the study group and received early nutritional intervention, including individualized nutrition counseling and oral nutritional supplements, from the initiation of CCRT to 2 weeks after its completion. The primary endpoint was the incidence of weight loss ≥5% during the CCRT. For comparison with the study group, a matched control group was retrieved from previous trials by the 1:1 propensity score matching method. **Results**: Sixty-seven patients were enrolled in the study group with a median follow-up of 52.4 months. Compared with the control group, the study group exhibited a lower incidence of weight loss ≥5% (*p* = 0.032), higher body mass index (*p* = 0.034) and prealbumin levels (*p* = 0.014) at the end of CCRT, as well as lower patient-generated subjective global assessments scores at the end of CCRT (*p* < 0.001) and 6 months after CCRT (*p* = 0.007). The study group also had a lower incidence of grade 2+ radiation pneumonitis (*p* = 0.023) and longer progression-free survival (13.5 vs. 11.3 months, *p* = 0.032). Patients who responded well to oral nutritional supplements had a higher Firmicutes/Bacteroidetes ratio at baseline (*p* = 0.036). **Conclusions**: Early nutritional intervention in LANSCLC patients undergoing CCRT improved nutritional status and reduced radiation pneumonitis. Gut microbiota was associated with the response to oral nutritional supplements.

## 1. Introduction

Globally, lung cancer ranks among the most prevalent and lethal malignancies. According to the 2020 Global Cancer Observatory (GLOBOCAN) report, it maintained its status as the primary contributor to cancer-related mortality, accounting for an estimated 1.8 million deaths, constituting 18% of all cancer fatalities [1]. Definitive concurrent chemoradiotherapy (CCRT) is a part of standardized treatment for unresectable locally advanced non-small cell lung cancer (LANSCLC). The common acute toxicities of CCRT include nausea, vomit, fatigue, and radiation esophagitis, which can impact patients’ appetites, potentially leading to weight loss and malnutrition during treatment [2]. Previous studies have substantiated that malnutrition risk in lung cancer patients ranges from 45% to 65%. It is noteworthy that a third of lung cancer patients experience malnutrition even before initiating treatment, with an additional third encountering significant weight loss during CCRT [3,4].

Malnutrition exerts a substantial influence on the prognosis of lung cancer patients. Luo J et al. conducted a retrospective analysis of 110 newly diagnosed NSCLC patients, revealing that nutritional status independently predicted the survival outcomes of NSCLC [5]. Similarly, Sánchez-Lara et al. identified malnutrition as an independent factor associated with the prognosis of NSCLC patients, emphasizing the need for prospective studies to explore the effects of diverse nutritional treatments on prognosis [6]. In our previous research, we observed a higher risk of radiation pneumonitis among malnourished LANSCLC patients undergoing CCRT [7]. Studies in patients with head and neck and gastrointestinal malignancies have demonstrated the potential of individualized nutrition counseling and interventions to enhance nutritional status and prognosis [8,9,10]. While oral nutritional supplements are typically favored for patients with intact gastrointestinal function, limited research pertains to patients with NSCLC, especially those receiving definitive CCRT. A study reported that nutrition counseling and/or oral nutritional supplements could improve the nutritional status and protein intake of NSCLC patients, though their impact on anti-tumor responses and survival outcomes remains unclear [11]. Patients with NSCLC undergoing CCRT often develop grade 2 or higher radiation-induced esophagitis within two weeks of treatment initiation, leading to dysphagia and weight loss. Dysphagia persists and worsens up to 2 weeks after CCRT completion, with subsequent healing occurring within 4–8 weeks. Notably, NSCLC patients may experience weight loss even before radiation-induced esophagitis occurs, and early weight loss is identified as a prognostic risk factor, suggesting the necessity of early nutritional intervention [12].

We conducted this prospective study to evaluate the impact of early nutritional intervention on nutritional status and survival outcomes in patients with LANSCLC undergoing CCRT. The intervention included personalized oral nutritional supplements and dietary counseling, initiated at the start of CCRT and continued until two weeks post-treatment. Nutritional and inflammatory indicators were monitored throughout the treatment period and up to six months following its completion. Additionally, we investigated the potential relationship between the outcomes of oral nutritional supplementation and gut microbiota composition.

## 2. Materials and Methods

### 2.1. Study Participants

This study (GASTO-1041) was designed as a single-arm, phase II trial with the following eligibility and exclusion criteria. Eligibility criteria were (1) unresectable stage IIIA to IIIC LANSCLC (as per the American Joint Committee on Cancer Tumor-Node-Metastasis staging system, eighth edition); (2) age between 18 and 75 years; (3) patient-generated subjective nutrition assessment (PG-SGA) of A or B; (4) Eastern Cooperative Oncology Group performance status (ECOG PS) of 0 to 1; (5) anticipated survival of more than 6 months; and (6) no contraindications to definitive CCRT. Exclusion criteria comprised: (1) significant intestinal dysfunction or inability to tolerate enteral nutrition; (2) severe gastrointestinal conditions such as persistent vomiting, gastrointestinal bleeding, or intestinal obstruction; and (3) severe malnutrition preventing tolerance of definitive CCRT. The study received approval from the review board of Sun Yat-Sen University Cancer Center and was conducted in accordance with the Declaration of Helsinki. All participants provided informed consent, and the study was registered on ClinicalTrials.gov (NCT03673657).

### 2.2. Treatment

Patients in this study received treatment following the institutional clinical protocol for definitive CCRT. A 4-dimensional computed tomography system (Brilliance CT Big Bore, Philips) was used to acquire images across 10 respiratory phases. A maximum intensity projection dataset was then reconstructed and utilized for tumor contouring and radiotherapy planning. The gross tumor volume (GTV) included the visible primary lesion and involved lymph nodes identified on CT or positron emission tomography scans. Internal GTV (IGTV) was directly delineated based on the maximum intensity projection images and then fine-tuned using CT scans from all respiratory phases. The clinical target volume (CTV) consisted of a 6 mm margin around IGTV and involved lymph node regions. The planning target volume (PTV)–GTV and PTV–CTV were produced by expanding IGTV and CTV with a 5 mm margin in all directions, respectively. We employed hypofractionated radiotherapy with a hypofractionated boost using intensity-modulated radiotherapy techniques [13,14]. Patients received hypofractionated radiotherapy to the PTV–GTV at a dose of either 51 Gy in 17 daily fractions or 40 Gy in 10 daily fractions, ensuring that at least 95% of the PTV volume was covered by the prescribed dose. Following treatment, a re-evaluation with contrast-enhanced chest and upper abdominal CT was performed to assess residual disease and patients’ physical conditions. A hypofractionated boost was administered to patients without disease progression or persistent grade ≥2 treatment-related toxicities. This boost targeted the residual tumor with 15 Gy in 5 daily fractions or 28 Gy in 7 daily fractions. Concurrent chemotherapy consisted of weekly docetaxel (25 mg/m^2^) and nedaplatin (25 mg/m^2^) during radiotherapy. No consolidative immunotherapy was used in this study.

### 2.3. Nutritional Intervention

Patients in the study group received early nutritional intervention, including individualized weekly nutrition counseling and oral nutritional supplements from the initiation of CCRT to 2 weeks after its completion. Weekly counseling sessions, lasting approximately 30 min, were conducted by both doctors and nurses. These sessions were separate from the standard weekly on-treatment monitoring appointments. While the standard monitoring addressed overall treatment progress and symptom management, the counseling sessions focused exclusively on nutritional education, personalized dietary advice, and support. Personalized dietary guidance offered specific recommendations regarding food selection, portion sizes, meal frequency, and nutritional targets, aiming to achieve a daily energy intake of around 30 kcal/kg and a protein intake of 1.2–1.5 g/kg. Additionally, oral nutritional supplements enriched with branched-chain amino acids were administered and tailored weekly during the counseling sessions, based on the patients’ updated nutritional assessments, weight trends, and dietary intake. To ensure adherence, the research team assessed the patients’ actual energy and nutrient intake weekly through food diaries and nutritional intake records and adjusted dietary recommendations accordingly.

### 2.4. Matched External Controls

A total of 71 LANSCLC patients who received hypofractionated radiotherapy and concurrent weekly chemotherapy, following the same treatment protocol, but without receiving early nutritional intervention, were selected from two independent prospective studies (NCT03900117 and NCT02573506) to serve as the control group. To minimize confounding factors, we performed a 1:1 propensity score matching process to achieve baseline comparability between the study and control groups. Propensity scores were derived through a logistic regression model incorporating multiple variables, including age, gender, ECOG PS, smoking history, diabetes history, clinical TNM stage (IIIA, IIIB, or IIIC), histologic subtypes (squamous cell carcinoma, adenocarcinoma, or others), and PG-SGA (A vs. B). Consequently, 67 patients were identified for the control group, each matched to a corresponding participant in the study group using a caliper width of 0.15. These control patients received routine clinical management without the provision of early individualized dietary guidance or nutrition counseling sessions. Patients experiencing nutritional risk were managed according to standard of care practices, which included additional dietary recommendations or support measures.

### 2.5. Data Acquisition

The primary outcome was the percentage of patients who experienced a weight reduction of ≥5% throughout the course of CCRT. Secondary endpoints included nutritional markers, quality of life (QoL), toxicities, and survival outcome. Nutrition-related parameters, including weight, body mass index (BMI), hemoglobin (HGB), serum albumin (ALB), pre-albumin (PA), lymphocyte (LY), and PG-SGA, were collected at baseline, at the end of CCRT, and 6 months after the completion of CCRT in both groups. Inflammation-related parameters, including C-reactive protein (CRP) and the neutrophil-to-lymphocyte ratio (NLR), were also assessed at these time points. To assess the QoL of patients in the study group, the European Organization for Research and Treatment of Cancer (EORTC) Quality of Life Questionnaire C30 (QLQ-C30) was applied. Treatment-related toxicity was evaluated by a radiation oncologist using the Common Terminology Criteria for Adverse Events version 5.0 (CTCAE version 5.0). Overall survival (OS) was measured from the initiation of CCRT until death from any cause or the last follow-up. Progression-free survival (PFS) was measured from the initiation of CCRT to disease progression, death, or the last follow-up.

### 2.6. Microbiome Analysis: Fecal DNA Extraction and 16S rRNA Sequencing

The analysis of gut microbiota was an exploratory endpoint of this study. Fecal samples were collected at four distinct time points: T0 (baseline), T1 (at the end of the first course of CCRT), T2 (at the start of the boost course of CCRT), and T3 (at the end of CCRT). To ensure the preservation of sample integrity, all fecal specimens were promptly frozen at −80 °C and treated with a DNA sample protector (Takara, 9750). Genomic DNA extraction was conducted on 100–200 mg of stool samples following the manufacturer’s instructions, employing the QIAamp PowerFecal DNA Kit (Qiagen, Hilden, Germany). The quality and integrity of extracted genomic DNA were evaluated using a NanoDrop 2000 spectrophotometer (Thermo Scientific, Waltham, MA, USA). PCR amplification targeted the V4–V5 regions of the bacterial 16S rRNA gene, and the purified amplicons were then sequenced using the Ion Torrent S5 XL platform (Thermo Fisher Scientific, Waltham, MA, USA). The FastQ files from the sequenced data were demultiplexed and subjected to quality filtering using DADA2 [15]. The QIIME2 pipeline was utilized to generate a feature table detailing the abundance of each assigned microbial taxon per sample [16].

### 2.7. Statistical Analysis

We hypothesized that the prevalence of weight loss ≥5% at the end of the treatment could be reduced from the 22% reported in previous studies [17] to 10% in the current study. To detect the expected improvement with 80% power at a one-tailed 0.05 significance level, 61 patients were needed. Accounting for a 10% dropout rate, the planned enrollment required 67 patients.

Categorical variables were analyzed using the χ^2^ test or Fisher’s exact test. Continuous variables were presented as mean (standard deviation) and median (interquartile range) and were compared using the t test and Mann–Whitney U test, respectively. For within-group comparisons across different time points, Friedman’s test was applied, and if statistical significance was observed, multiple comparisons were performed using Wilcoxon’s signed-rank test with Bonferroni’s adjustment. OS and PFS for both groups were estimated using Kaplan–Meier curves, with differences compared via the log-rank test. Gut microbiota analysis included Wilcoxon rank-sum tests for alpha diversity (Shannon index) and taxonomic composition, while beta diversity was assessed through principal coordinate analysis (PCoA) based on Bray–Curtis dissimilarities. Data analysis was performed with SPSS version 26.0 (IBM Corp., Armonk, NY, USA), and a two-tailed *p*-value < 0.05 was considered statistically significant, with Bonferroni adjustment applied to *p*-values.

## 3. Results

### 3.1. Patient Baseline Demographics

**Figure 1:**
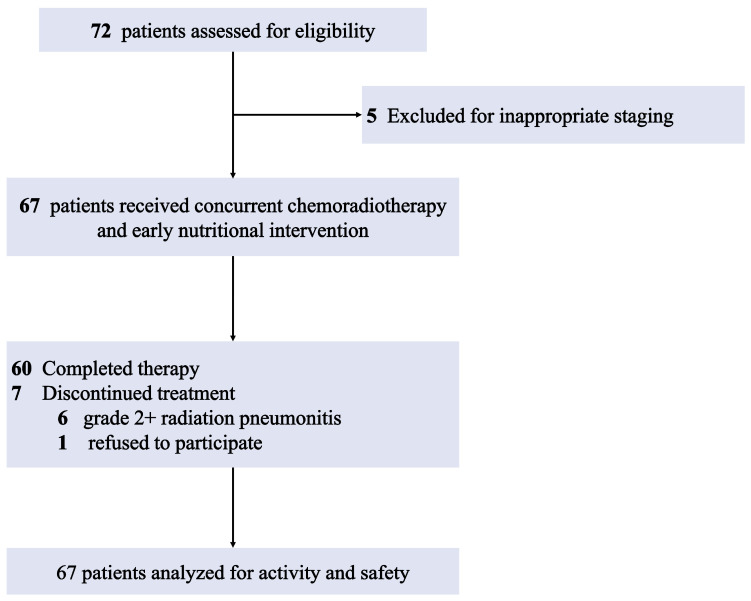
CONSORT diagram of the trial.

A total of 72 patients provided their consent to participate in this study between 20 September 2018 and 16 March 2020. After excluding five patients for incorrect staging, 67 patients were retained for analysis (Figure 1). The baseline characteristics of the included patients are presented in Table 1. Among the participants, the median age was 58 years, with 53 (79.1%) being male, 37 (55.2%) having an ECOG PS of 1, and 45 (67.2%) having a history of smoking. The distribution of histologic subtypes included 32 cases (47.8%) of squamous cell carcinoma and 23 cases (34.3%) of adenocarcinoma. Disease staging revealed 24 patients (35.8%) with stage IIIA, 26 (38.8%) with stage IIIB, and 17 (25.4%) with stage IIIC. Nine patients (13.4%) had diabetes mellitus as a comorbidity. Approximately 40 patients (59.7%) had a PG-SGA grade of A, and the mean BMI was 23.79 ± 2.79 kg/m^2^. After matching, the control group consisted of 67 patients, and there were no significant differences in the baseline characteristics between the two groups.

### 3.2. Treatment Details

In the study group, the median total radiation dose administered was 68 Gy (range, 40–68). The majority of patients (89.6%, *n* = 60) completed both courses of CCRT according to the protocol. Seven patients (10.4%) discontinued treatment, with reasons including grade 2 or higher radiation pneumonitis (*n* = 6) and patient refusal (*n* = 1). In the control group, the median total radiation dose was similarly 68 Gy (range, 40–68). A slightly lower proportion of patients (79.1%, *n* = 53) completed both CCRT courses, while 14 patients discontinued for reasons such as radiation pneumonitis (*n* = 7), disease progression (*n* = 3), patient refusal (*n* = 3), and esophagitis (*n* = 1). The dosimetric parameters were consistent across both groups (Supplementary Table S1).

### 3.3. Changes of Nutritional and Inflammatory Parameters in the Two Groups

The study group exhibited a significantly lower incidence of weight loss ≥5% during CCRT, with only 9.0% (6/67) of patients experiencing this level of weight reduction, compared to 22.4% (15/67) in the control group (*p* = 0.032). Weight and BMI exhibited a consistent increase from baseline to 6 months after CCRT in the study group, while they decreased during CCRT and showed slight recovery 6 months after CCRT in the control group. Notably, at the end of CCRT, weight, BMI, and the levels of PA were significantly higher in the study group compared to the control group (*p* = 0.024, *p* = 0.034, and *p* = 0.014, respectively). In terms of the inflammatory status of the patients, CRP and neutrophil-to-lymphocyte ratio (NLR) consistently increased from baseline to 6 months after CCRT. Specifically, at the end of CCRT, NLR was lower in the study group compared to the control group (*p* = 0.011). Further details are provided in Table 2.

**Figure 2:**
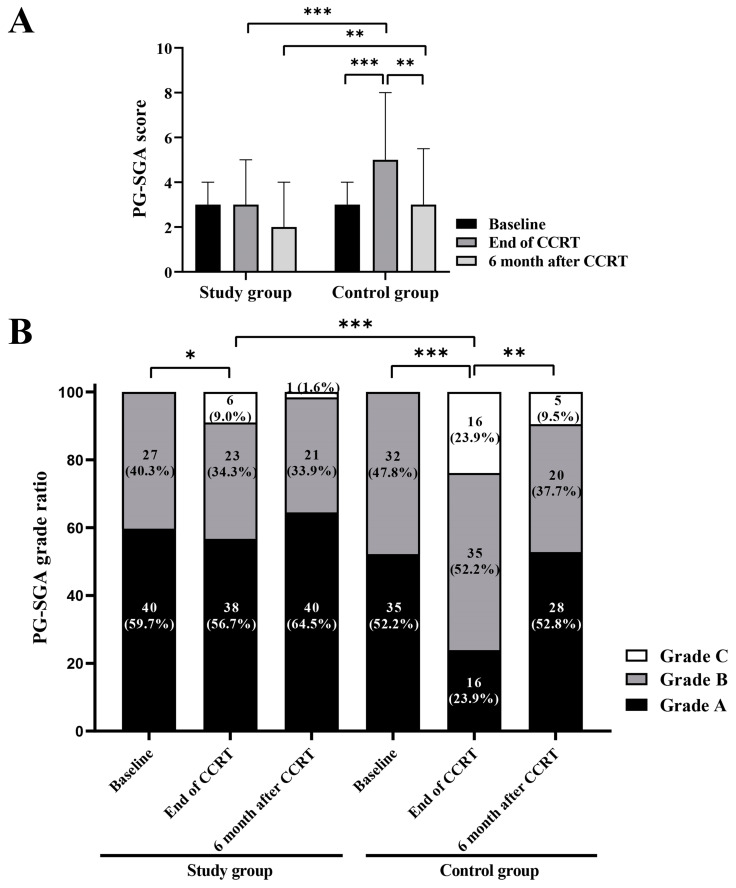
The comparison of patient-generated subjective nutrition assessment (PG-SGA) scores and grades between the study and control groups. (**A**) The median PG-SGA scores of the two groups at baseline, at the end of the concurrent chemotherapy (CCRT), and 6 months after CCRT. (**B**) The PG-SGA grade composition of the two groups at baseline, at the end of the CCRT, and 6 months after CCRT. * *p* < 0.05; ** *p* < 0.01; *** *p* < 0.001.

The median PG-SGA scores were 3 (interquartile ratio [IQR], 3–4), 3 (IQR, 1–5), and 2 (IQR, 1–4) at baseline, end of the CCRT, and 6 months after CCRT in the study group, while they were 3 (IQR, 3–4), 5 (IQR, 4–8), and 3 (IQR, 3–5.5) in the control group, respectively (Figure 2A). The PG-SGA scores were significantly lower in the study group at the end of CCRT (*p* < 0.001) and 6 months after CCRT (*p* = 0.007). Furthermore, the PG-SGA scores at the end of CCRT in the control group significantly differed from those at baseline (*p* < 0.001) and those at 6 months after CCRT (*p* = 0.001), while no significant differences were observed within the study group. Both the study and control groups displayed a decrease in the proportion of PG-SGA grade A and an increase in grade C at the end of CCRT, with a subsequent recovery at 6 months after CCRT (Figure 2B). Notably, at the end of CCRT, 6 patients (9.0%) in the study group and 16 patients (23.9%) in the control group were assessed as grade C. The distribution of PG-SGA grades significantly differed between the two groups at the end of CCRT (*p* < 0.001).

### 3.4. QoL Scores of the Study Group

The findings from the health-related QoL analysis, conducted using the EORTC QLQ-C30 (ver. 3.0), are presented in Supplementary Table S2. In the study group, the overall QoL score showed a significant decrease at the end of CCRT, but recovered to baseline levels within 6 months after CCRT (*p* < 0.001). Regarding functional scales, each category’s score declined at the end of CCRT, but improved in the 6 months following CCRT. Notably, physical functioning (*p* = 0.031), emotional functioning (*p* < 0.001), and social functioning (*p* < 0.001) displayed significant improvements 6 months after CCRT compared to the end of CCRT. In the symptomatic scales, the scores related to symptoms increased after CCRT, but decreased in the 6 months after CCRT. Specifically, symptoms such as fatigue, gastrointestinal symptoms (nausea and vomiting, appetite loss), pain, insomnia, and financial difficulties exhibited significant changes from baseline to 6 months after CCRT.

### 3.5. Treatment Toxicity

The treatment-related adverse events (AEs) are presented in Supplementary Table S3. The incidence of grade ≥2 radiation esophagitis was similar between the study group and the control group (50.7% vs. 59.7%, *p* = 0.297). Likewise, the occurrence of grade ≥3 hematologic AEs, including leukopenia (4.5% vs. 4.5%), neutropenia (3.0% vs. 3.0%), lymphopenia (73.1% vs. 73.1%), anemia (0 vs. 1.5%), and thrombocytopenia (1.5% vs. 1.5%), did not significantly differ between the two groups. However, grade 2 or higher radiation pneumonitis occurred notably less frequently in the study group than in the control group (20.9% vs. 38.8%, *p* = 0.023).

### 3.6. Survival Outcome

**Figure 3:**
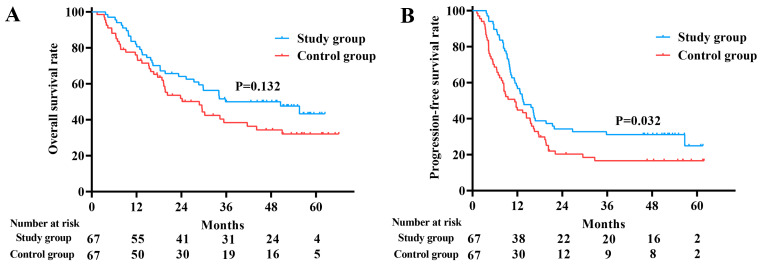
The overall survival rate (**A**) and progression-free survival rate (**B**) of the study and control groups.

The median follow-up duration in the study group was 52.4 months (95% confidence interval [CI]: 50.5–54.3). The median OS and PFS were 35.8 months (95% CI: 11.4–60.2) and 13.5 months (95% CI: 9.5–17.6), respectively. For the control group, the median follow-up duration was 54.8 months (95% CI: 47.9–61.8), with corresponding OS and PFS of 28.8 months (95% CI: 17.5–40.1) and 11.3 months (95% CI: 7.2–15.4), respectively. PFS was significantly prolonged in the study group compared to the control group (*p* = 0.032), whereas OS showed no statistically significant difference between the two groups (*p* = 0.132) (Figure 3A,B).

### 3.7. Microbiota Profiling and Exploratory Insights

**Figure 4:**
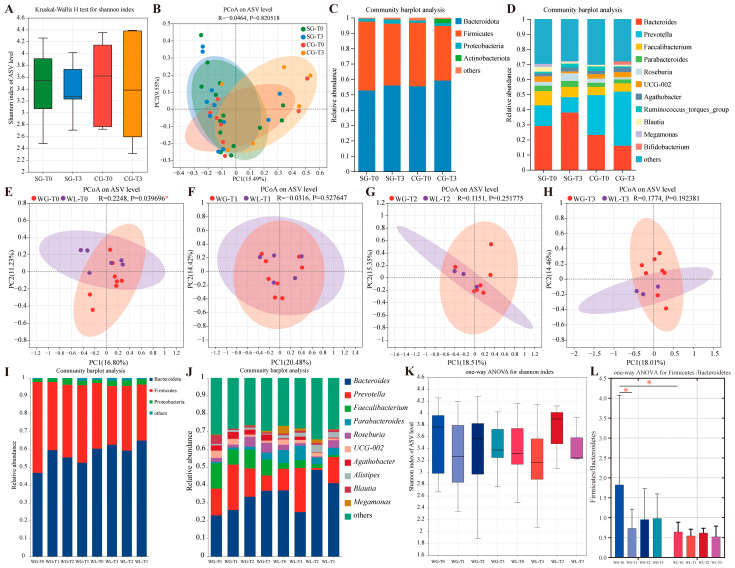
Analysis of the gut microbiota. (**A**) Alpha diversity (Shannon index) on amplicon sequence variant (ASV) level of the study group (SG) and control group (CG) at T0 and T3. (**B**) Principal coordinates analysis (PCoA) of the community structure between the two groups at T0 and T3. (**C**,**D**) The phylum and genus composition of the two groups at T1 and T3, respectively. (**E**–**H**) PCoA of the community structure between the weight gain (WG) subgroup and the weight loss (WL) subgroup at each time point. (**I**,**J**) The phylum and genus composition of the WG subgroup and the WL subgroup at T1 to T3, respectively. (**K**) Alpha diversity (Shannon index) of the two subgroups at T0 to T3. (**L**) The Firmicutes/Bacteroidetes (F/B) ratio of the two subgroups at each timepoint. Timepoints: T0, baseline; T1, at the end of the first course of CCRT; T2, at the start of the boost course of CCRT; T3, at the end of CCRT. * *p* < 0.05.

To explore the impact of oral nutritional supplements on gut microbiota, 14 patients from the study group and 8 patients from the control group were analyzed. There were no significant differences in the clinical characteristics between the two groups (Supplementary Table S4). No significant differences were observed in alpha diversity (Shannon index) or community structures between the two groups at the T1 or T3 timepoints (Figure 4A,B). The phylum and genus composition of the two groups at T1 and T3 timepoints are presented in Figure 4C,D. The relative abundance of *Bacteroides* increased, while *Prevotella* decreased in the study group from T0 to T3, with the opposite trend observed in the control group.

To investigate the differences in gut microbiota between patients who responded well to oral nutritional supplements and those who did not, the 14 patients in the study group were divided into two subgroups: 8 patients were defined as the weight loss subgroup, while the others belonged to the weight gain subgroup. The comparison of clinical nutritional parameters between these two subgroups is presented in Supplementary Table S5. Except for the significant changes in weight and BMI between the two subgroups, the changes in PG-SGA scores were significantly improved in the weight gain subgroup (*p* = 0.020). Principal coordinates analysis at each timepoint revealed that community structures were significantly different between the two subgroups at baseline (*p* = 0.040) (Figure 4E–H). The primary phylum and genus composition of the two subgroups is demonstrated in Figure 4I,J at each time point. The Shannon index did not exhibit a significant difference between the two subgroups at the four time points (Figure 4K). The analysis of the Firmicutes/Bacteroidetes (F/B) ratio revealed that the weight gain subgroup had a significantly higher F/B ratio at baseline (*p* = 0.036) compared with the weight loss subgroup, and the F/B ratio of this subgroup significantly declined at the completion of the first course of CCRT (*p* = 0.044) and partially recovered during the subsequent treatment (Figure 4L).

## 4. Discussion

Although the symptomatic effects of radiation injury and their impact on nutrition have been well-recognized, the advantages of early nutritional intervention on patient outcomes have yet to be fully explored. To address this, we conducted a single-arm phase II study aimed to evaluate the effectiveness and importance of early nutritional intervention in LANSCLC patients undergoing CCRT. In comparison to a control group receiving the same chemoradiation scheme without specific dietary interventions or nutrition counseling, our study demonstrated that early nutritional intervention could reduce the occurrence of treatment-associated weight loss ≥5%, enhance nutritional status and quality of life, and lead to favorable clinical outcomes.

There is currently a scarcity of research on the effects of nutritional interventions in patients with NSCLC. In one randomized study involving 24 lung cancer patients, participants received either intensive dietary counseling from the start of treatment until six weeks after its completion or standard care [18]. The intervention group demonstrated notable improvements in body weight, fat-free mass, and both physical and functional well-being. Another secondary analysis of a prospective randomized study including 113 lung cancer patients showed that individualized nutritional support, compared to routine hospital nutrition without personalization, significantly lowered mortality risk and enhanced functional outcomes and quality of life in nutritionally at-risk patients [19]. These results align with our findings and emphasize the value of timely and personalized nutritional strategies for NSCLC patients undergoing CCRT.

The primary endpoint of this study was weight loss during CCRT, a critical indicator of nutritional decline. Previous investigations have reported a prevalence of weight loss ≥5% in approximately 22–34% of LANSCLC patients undergoing CCRT [3,17,20,21]. In our trial, we observed weight loss ≥5% in 10.0% of patients in the study group and 29.2% in the control group. It is worth noting that the incidence might have been underestimated due to missing weight data for a subset of patients, particularly those who discontinued treatment. Numerous studies have linked BMI and weight loss to survival outcomes. Patel et al. discovered that NSCLC patients who gained weight during CCRT exhibited improved OS and PFS [22]. Similar findings were reported in a pooled analysis of patients with NSCLC conducted by Le-Rademacher et al. They revealed that a weight loss of 2% or more was associated with inferior OS when compared to weight gain or weight loss less than 2% [23]. Oswalt et al. also noted that a weight loss ≥5% was associated with shorter OS, with the percentage of weight loss exerting a more significant influence on survival than BMI, especially in the NSCLC subgroup [21]. We identified that patients who received early nutritional intervention displayed extended PFS in our study. The extended PFS in the study group was potentially attributed to a higher completion rate of CCRT. Additionally, well-nourished patients tended to have more favorable tumor microenvironment and therapy sensitivity. Effective control of weight loss throughout and following CCRT requires a comprehensive approach, including evaluation of nutritional needs, individualized dietary guidance, patient education, continuous monitoring of adherence, and timely intervention for symptom management [10].

Early nutritional intervention appeared to effectively enhance the nutritional status of patients undergoing CCRT. In comparison to the control group, PG-SGA scores were significantly lower at the end of CCRT and 6 months after CCRT and remained relatively stable during the treatment and subsequent 6 months. Despite the intervention, 9.0% of patients in the study group and 23.9% in the control group still had PG-SGA scores ≥9, a threshold linked to an approximately 80% risk of malnutrition across cancer populations [24]. Additionally, early nutritional support did not lead to significant changes in blood-based indicators of nutritional status. They exhibited a decrease during CCRT and subsequently recovered in the 6 months after CCRT in the two groups, with the exception of PA. PA followed an opposite trend, being significantly higher at the end of CCRT in the study group. PA is a sensitive marker for assessing the state of malnutrition and has been validated as a significant prognostic factor for lung cancer [25]. A persistent and severe inflammatory status can result in an energy deficit and subsequent weight loss. CRP and NLR, as markers of inflammation, increased at the end of CCRT and did not return to the level at diagnosis after 6 months. Notably, the NLR level at the end of CCRT was significantly lower in the study group compared to the control group. This observation suggested that early nutritional intervention may alleviate the inflammatory response, while radiation-related inflammation may contribute to maintaining CRP levels after treatment.

QoL evaluation—capturing patients’ perspectives on disease burden, treatment effects, expectations, and overall satisfaction—serves as a crucial and independent endpoint in clinical research, often regarded as the gold standard. In our study, global health status and single-item scores deteriorated during CCRT and subsequently improved within 6 months in the study group. Fatigue, gastrointestinal symptoms (nausea and vomiting, appetite loss), and pain exhibited significant changes from baseline to 6 months after CCRT. Fatigue is a prevalent issue experienced by cancer patients during treatment, significantly affecting daily activities and diminishing overall QoL. Gastrointestinal symptoms, including nausea, vomiting, appetite loss, and swallowing pain, could result in weight loss directly. However, QoL assessment data from the control group were unavailable, emphasizing the need for further randomized controlled trials to elucidate the effects of early nutritional intervention on the quality of life.

It is well-established that the severity of radiotherapy-induced toxicity depends on tumor histology, total dose, fractionation, radiation volume, repair mechanisms, and concurrent chemotherapy [10]. Our study revealed that the incidence of grade ≥2 radiation pneumonitis was significantly reduced in the study group, while the incidence of grade ≥2 radiation esophagitis and grade ≥3 hematologic AEs exhibited no differences between the two groups. The results indicated that early nutritional intervention might improve functional and mobility status through reducing the incidence of pneumonitis, which was in accordance with a previous study indicating that malnourished LANSCLC patients had a higher risk of radiation pneumonitis during CCRT [7]. More than half of the patients in both groups had grade ≥2 radiation esophagitis, which was one of the main reasons for weight loss. Interestingly, a study reported that weight loss during CCRT for NSCLC patients started early, preceding the onset of esophagitis, underscoring the importance of early and comprehensive nutritional rehabilitation during the treatment [20].

The interactions among LANSCLC, nutrition, and gut microbiota are intricate and multifaceted. Various dietary components could cause the change of gut microbiota. Research has indicated that protein consumption can increase the diversity of gut microbiota, carbohydrates can lead to the intensive growth of *Bacteroides*, and polyunsaturated fats can foster the growth of *Ruminococcus* [26]. A study demonstrated differences in the community structure of gut microbiota between cachectic and non-cachectic cancer patients, with cachectic patients showing a higher abundance of Proteobacteria and *Veillonella* [27]. In our study, we did not find significant changes in the alpha or beta diversity of gut microbiota, but we observed alterations in the relative abundance of *Bacteroides* and *Prevotella* between the two groups. Further analysis based on different responses to oral nutritional supplements suggested that the community structure and a higher F/B ratio at baseline might be associated with the favorable nutritional intervention outcomes. A recent systematic review indicated a relationship between an increased F/B ratio and obesity in the majority of studies [28], which aligns with our study results. Regulating the F/B ratio during early nutritional intervention in cancer patients is a consideration for future research.

Although our results are promising for the application of early nutritional intervention at the onset of CCRT in clinical practice, our study has several limitations. Firstly, it was a phase II single-arm study with a small sample size involving matched controls. The non-randomized nature of the historical control group will be clearly acknowledged. Larger randomized controlled trials are needed to validate our findings. Secondly, the absence of data on metabolic factors may have limited the detection of other relationships. Thirdly, the percentage of weight loss is a general parameter that does not specifically define lean body mass and can be influenced by various factors, including edema, ascites, and organ volume. More specific biomarkers are required to accurately reflect nutritional status. Fourthly, the exploratory results regarding the gut microbiota need validation in a larger population. Finally, no consolidative immunotherapy was used in this study, as the start time of the study was earlier than the approval of consolidative immunotherapy in our country. This strategy shows potential and warrants further investigation when combined with CCRT and subsequent immunotherapy in patients with LANSCLC.

## 5. Conclusions

In conclusion, for patients with LANSCLC receiving CCRT, early nutritional intervention—comprising dietary counseling and oral nutritional supplementation—beyond standard dietary intake may contribute to improved clinical outcomes. This approach was associated with increased body weight, enhanced PG-SGA scores, reduced treatment-related toxicity, and better survival. Gut microbiota, in terms of Firmicutes/Bacteroidetes ratio at baseline, was associated with the responses to oral nutritional supplements.

## Figures and Tables

**Table 1:** Patient baseline demographics.

Characteristic	Study Group	Control Group	*p* Value
	(*n* = 67)	(*n* = 67)	
Age, yrs			
Median (IQR)	58 (51–65)	58 (52–67)	0.561
Gender, *n* (%)			
Male	53 (79.1)	50 (74.6)	0.539
Female	14 (20.9)	17 (25.4)	
ECOG, *n* (%)			
0	30 (44.8)	34 (50.7)	0.489
1	37 (55.2)	33 (49.3)	
Smoking history, *n* (%)			
Smoker	45 (67.2)	41 (61.2)	0.471
Non-smoker	22 (32.8)	26 (38.8)	
Diabetes mellitus, *n* (%)			
Yes	9 (13.4)	8 (11.9)	0.795
No	58 (86.6)	59 (88.1)	
Histology, *n* (%)			
Adenocarcinoma	23 (34.3)	23 (34.3)	0.971
Squamous cell carcinoma	32 (47.8)	33 (49.3)	
Others	12 (17.9)	11 (16.4)	
Disease stage, *n* (%)			
IIIA	24 (35.8)	25 (37.3)	0.938
IIIB	26 (38.8)	24 (35.8)	
IIIC	17 (25.4)	18 (26.9)	
PG-SGA, *n* (%)			
A	40 (59.7)	35 (52.2)	0.384
B	27 (40.3)	32 (47.8)	
BMI, kg/m^2^			
Mean (SD)	23.79 (2.79)	23.65 (2.97)	0.772

Abbreviations: BMI, body mass index; IQR, interquartile ratio; PG-SGA, patient-generated subjective global assessment; SD, standard deviation.

**Table 2:** The nutrition parameters of two groups across different timepoints.

Variables		Baseline	End of CCRT	6 Months After CCRT
		Median (IQR)/Mean (SD)		
Weight (kg)	Study group	62.61 (8.58)	63.67 (8.18)	63.73 (7.94)
	Control group	62.00 (9.53)	60.00 (8.52)	60.35 (6.85)
	*p* value	0.708	0.024	0.082
BMI (kg/m^2^)	Study group	23.79 (2.79)	24.13 (2.75)	24.38 (2.80)
	Control group	23.65 (2.97)	23.03 (2.51)	23.27 (2.44)
	*p* value	0.772	0.034	0.106
ALB (g/L)	Study group	43.68 (2.90)	41.24 (3.49)	43.23 (3.57)
	Control group	43.19 (3.50)	41.21 (2.24)	42.02 (3.94)
	*p* value	0.381	0.965	0.145
PA (mg/dL)	Study group	26.53 (4.68)	28.59 (5.04)	26.92 (4.92)
	Control group	25.30 (5.38)	25.86 (5.92)	25.49 (6.51)
	*p* value	0.189	0.014	0.352
LY × 10^9^/L	Study group	1.77 (0.58)	0.58 (0.21)	1.26 (0.53)
	Control group	1.71 (0.64)	0.52 (0.22)	1.16 (0.59)
	*p* value	0.600	0.152	0.435
HGB (g/L)	Study group	130 (19)	127 (15)	135 (15)
	Control group	129 (16)	125 (11)	131 (20)
	*p* value	0.671	0.391	0.364
NLR	Study group	2.07 (1.58–3.28)	6.34 (4.70–8.53)	3.42 (2.29–5.01)
	Control group	2.52 (1.85–3.83)	8.00 (5.96–10.66)	4.17 (2.44–6.51)
	*p* value	0.062	0.011	0.285
CRP (mg/L)	Study group	2.93 (1.60–9.00)	4.27 (2.12–10.74)	7.12 (2.84–23.29)
	Control group	4.01 (1.47–17.49)	5.38 (2.88–13.82)	6.46 (2.52–24.28)
	*p* value	0.368	0.185	0.910

Abbreviation: ALB, albumin; BMI, body mass index; CCRT, concurrent chemoradiotherapy; CRP, C-reaction protein; HGB, hemoglobin; IQR, interquartile ratio; LY, lymphocyte; NLR, neutrophil-to-lymphocyte ratio; PA, prealbumin; SD, standard deviation.

## Data Availability

Individual data will be made available following publication by reasonable request to the corresponding author due to privacy concerns. The study protocol is available in the Supplementary Materials.

## Supplementary Materials

The following supporting information can be downloaded at https://www.mdpi.com/article/10.3390/nu17081389/s1. Table S1. Dosimetric parameters of patients in two cohorts. Table S2. The QoL scores at baseline, the end of CCRT, and 6 months after CCRT in the study group. Table S3. Comparison of treatment-related toxicities between the two groups. Table S4. Baseline characteristics of patients. Table S5. Baseline characteristics, clinical nutritional parameters before and after CCRT between two subgroups. File S1: Study Protocol.
