# Supplementary material for: Early Nutritional Intervention in Patients with Non-Small Cell Lung Cancer Receiving Concurrent Chemoradiotherapy: A Phase II Prospective Study
Source: Nutrients. 2025 Apr 21;17(8):1389. doi: 10.3390/nu17081389 (PMC12030435; doi:10.3390/nu17081389)
Supplement: Supplementary file 1 [file nutrients-17-01389-s001.zip › Supplementary Material.pdf]

Supplementary Table S1. Dosimetric parameters of patients in two cohorts.

Parameters	Study group (N=67)	Control group (N=67)	*P* value
GTV volume, median (cm^3, IQR)	100.20 (67.24-146.89)	105.80 (84.51-145.99)	0.652
Lungs V_{20}, median (%, IQR)	25.62 (19.05-29.23)	27.98 (18.32-30.03)	0.717
Mean dose of lungs, median (Gy, IQR)	15.48 (13.45-16.85)	16.47 (14.35-18.06)	0.307
Heart V_{30}, median (%, IQR)	9.37 (5.54-13.14)	11.54 (6.99-13.94)	0.398
Mean dose of heart, median (Gy, IQR)	10.24 (7.40-12.92)	12.14 (7.88-13.82)	0.272
Esophagus V_{60}, median (%, IQR)	1.52 (0-2.34)	1.40 (0.48-4.06)	0.732
Mean dose of esophagus, median (Gy, IQR)	28.66 (23.54-30.72)	26.86 (25.67-29.98)	0.482
Maximum dose of esophagus, median (Gy, IQR)	60.86 (57.14-62.79)	60.77 (56.20-61.99)	0.448
Maximum dose of spinal cord, median (Gy, IQR)	28.58 (22.79-36.31)	31.50 (24.53-33.95)	0.606

Abbreviations: GTV, gross target volume; IQR, interquartile range; V_{20}, the percentage of the total organ volume receiving ≥20 Gy; V_{30}, the percentage of the total organ volume receiving ≥30 Gy, V_{60}, the percentage of the total organ volume receiving ≥60 Gy.

Supplementary Table S2. The QoL scores at baseline, the end of CCRT, and 6 months after CCRT in the study group.

Subscale	Baseline	End of CCRT	6 months after CCRT	All 3 Times*	Baseline vs. end#	Baseline vs. 6 months#	End vs. 6 months#
	Mean±SD			P-value			
**Global health status**	80.35±12.28	73.26±9.68	84.27±10.25	**<0.001**	**<0.001**	0.052	**<0.001**
**Functional scales**							
Physical functioning	93.13±13.63	90.45±16.67	94.84±11.09	**<0.001**	>0.999	0.318	**0.031**
Role functioning	96.27±9.09	93.28±14.52	97.04±8.28	**0.005**	>0.999	>0.999	0.579
Emotional functioning	90.30±16.39	83.46±22.87	94.89±9.82	**<0.001**	0.130	0.066	**<0.001**
Cognitive functioning	89.80±18.34	87.06±24.24	88.71±18.79	0.743			
Social functioning	76.87±23.20	69.15±20.36	82.80±16.24	**<0.001**	**0.036**	0.074	**<0.001**
**Symptom scales**							
Fatigue	6.97±13.52	14.43±19.82	7.71±11.66	**<0.001**	0.059	>0.999	0.104
Nausea and vomiting	2.99±9.14	7.96±15.17	3.76±10.20	**<0.001**	0.264	>0.999	0.492
Pain	3.48±12.15	8.46±16.51	3.76±9.74	**0.004**	0.217	>0.999	0.579
Dyspnea	12.44±20.79	14.43±19.44	11.83±20.11	0.208			
Insomnia	9.45±19.07	9.45±18.17	5.91±12.84	**0.014**	>0.999	0.844	0.844
Appetite loss	4.98±14.51	11.44±17.93	6.45±14.58	**0.002**	0.264	>0.999	0.492
Constipation	3.48±10.27	3.98±10.89	2.69±9.15	0.174			
Diarrhea	1.49±6.95	2.49±8.83	1.61±7.21	0.607			
Financial difficulties	11.44±17.93	15.92±21.21	7.53±14.05	**<0.001**	0.676	0.676	**0.046**

* Related-samples Friedman's two-way analysis of variance by ranks.

# Bonferroni adjusted.

Abbreviation: CCRT, concurrent chemoradiotherapy; QoL, quality of life; SD, standard deviation.

Supplementary Table S3. Comparison of treatment-related toxicities between the two groups.

	Study group (n=67)					Control group (n=67)				
Toxicities, n (%)	<G2	G2	G3	G4	G5	<G2	G2	G3	G4	G5
Leukopenia	50 (74.6)	14 (20.9)	3 (4.5)	0	0	51 (76.1)	13 (19.4)	3 (4.5)	0	0
Neutropenia	59 (88.0)	6 (9.0)	1 (1.5)	1 (1.5)	0	59 (88.0)	6 (9.0)	2 (3.0)	0	0
Lymphopenia	3 (4.5)	15 (22.4)	40 (59.7)	9 (13.4)	0	5 (7.5)	13 (19.4)	37 (55.2)	12 (17.9)	0
Anemia	58 (86.6)	9 (13.4)	0	0	0	54 (80.6)	12 (17.9)	1 (1.5)	0	0
Thrombocytopenia	61 (91.0)	5 (7.5)	1 (1.5)	0	0	63 (94.0)	3 (4.5)	1 (1.5)	0	0
Esophagitis	33 (49.3)	26 (38.8)	8 (11.9)	0	0	27 (40.3)	30 (44.8)	10 (14.9)	0	0
Radiation pneumonitis	53 (79.1)	11 (16.4)	1 (1.5)	0	2 (3.0)	41 (61.2)	21 (31.3)	3 (4.5)	0	2 (3.0)

Supplementary table S4. Baseline characteristics of patients.

Characteristic	Study group (n=14)	Control group (n=8)	*P* value
Age, y			
Median (IQR)	58 (54-67)	58 (48-67)	0.868
Gender, n (%)			
Male	12 (85.7)	8 (100)	0.515
Female	2 (14.3)	0 (0)	
ECOG, n (%)			
0	4 (28.6)	3 (37.5)	>0.999
1	10 (71.4)	5 (62.5)	
Smoking history, n (%)			
Smoker	11 (78.6)	7 (87.5)	>0.999
Non-smoker	3 (21.4)	1 (12.5)	
Diabetes mellitus, n (%)			
Yes	1 (7.1)	1 (12.5)	>0.999
No	13 (92.9)	7 (87.5)	
Histology, n (%)			
Adenocarcinoma	6 (42.9)	4 (50.0)	0.822
Squamous cell carcinoma	7 (50.0)	3 (37.5)	
Others	1 (7.1)	1 (12.5)	
Disease stage, n (%)			
IIIA	3 (21.4)	2 (25.0)	0.769
IIIB	9 (64.3)	4 (75.0)	
IIIC	2 (14.3)	2 (25.0)	
PG-SGA, n (%)			
A	9 (64.3)	3 (37.5)	0.378
B	5 (35.7)	5 (62.5)	
BMI, kg/m^2			
Mean (SD)	23.19 (2.97)	25.44 (3.48)	0.139

Abbreviations: BMI, body mass index; IQR, interquartile ratio; PG-SGA, patient-generated subjective global assessment; SD, standard deviation.

Supplementary table S5. Baseline characteristics, clinical nutritional parameters before and after CCRT between two subgroups.

Characteristic	Weight gain group (n=8)	Weight loss group (n=6)	*P* value
Age, y			
Median (IQR)	60 (52-66)	57 (54-67)	0.755
Gender, n (%)			
Male	7 (87.5)	5 (83.3)	>0.999
Female	1 (12.5)	1 (16.7)	
ECOG, n (%)			
0	2 (25.0)	2 (33.3)	>0.999
1	6 (75.0)	4 (66.7)	
Smoking history, n (%)			
Smoker	7 (87.5)	4 (66.7)	0.538
Non-smoker	1 (12.5)	2 (33.3)	
Diabetes mellitus, n (%)			
Yes	0 (0)	1 (16.7)	0.429
No	8 (100)	5 (83.3)	
Δ PG-SGA	-2 (-4~-1)	2 (-1~6)	**0.020**
Δ weight (kg)	1.90±0.93	-2.36±1.84	**0.004**
Δ BMI (kg/m^2)	0.72±0.37	-0.84±0.61	**<0.001**
Δ ALB (g/L)	-1.74±5.04	-3.84±2.80	0.416
Δ PA (mg/dl)	2.40±5.83	-0.45±4.30	0.411
Δ LY ×10^9/L	-1.07±0.48	-1.18±0.98	0.788
Δ HGB (g/L)	2±12	-8±10	0.169

Δ (change) = (end of CCRT data) − (baseline data).

Abbreviations: ALB, albumin; BMI, body mass index; CCRT, concurrent chemoradiotherapy; HGB, hemoglobin; IQR, interquartile ratio; LY, lymphocyte; PA, prealbumin; PG-SGA, patient-generated subjective global assessment.
