# Supplementary material for: Early Nutritional Intervention in Patients with Non-Small Cell Lung Cancer Receiving Concurrent Chemoradiotherapy: A Phase II Prospective Study
Source: Nutrients. 2025 Apr 21;17(8):1389. doi: 10.3390/nu17081389 (PMC12030435; doi:10.3390/nu17081389)
Supplement: Supplementary file 1 [file nutrients-17-01389-s001.zip › File S1-Study Protocol.pdf]

Early nutritional intervention in patients with locally advanced non-small cell lung cancer receiving concurrent chemoradiotherapy: a single-arm phase II prospective study

Document type:	Clinical Trial Protocol
Development phase:	II

Table of contents

Table of contents 2
Protocol synopsis 4
1 Introduction 6
2 Study objectives 7
2.1 Primary endpoint 7
2.2 Secondary endpoints 7
2.3 Exploratory analysis 8
3 Investigational plans 8
3.1 Study design 8
3.2 Research progress and completion time 8
3.3 Comparison with historical control group 8
4 Population 9
4.1 Sample size 10
4.2 Inclusion criteria 10
4.3 Exclusion criteria 10
4.4 Premature patient withdrawal 10
5 Treatment 11
5.1 Nutrition intervention 11
5.2 Radiotherapy 12
5.2.1 Postural immobilization 12
5.2.2 Definition of target area 12
5.2.3 Radiotherapy plan 13
5.2.4 Dosage prescription 13
5.2.5 Organ at risk 14
5.2.6 Delivery of treatment 15
5.2.7 Quality control 15
5.3 Concurrent chemotherapy 16
6 Adverse events and treatment plan adjustment 16
6.1 Definition 16
6.1.1 Adverse events (AE) 16
6.1.2 Serious adverse events (SAE) 17
6.2 Toxicity of radiotherapy and management of radiation-related side effects 18
6.3 Toxicity of chemotherapy and chemotherapy dose adjustment 19
7 Visit schedule and assessments 20
7.1 The baseline assessment 20
7.2 The assessment during the treatment 21
7.3 The assessment after the completion of treatment 21

7.4 Quality of life score 22
7.5 Follow up 22
8 Evaluation of the research results 23
8.1 Efficacy 23
8.1.1 Incidence of weight loss 23
8.1.2 Nutritional status 23
8.1.3 Survival outcomes 23
8.2 Safety assessment 23
8.2.1 Overall safety 23
8.2.2 Laboratory evaluations 23
9 Statistics 24
9.1 Analysis sets 24
9.2 Patient demographics and other baseline characteristics 24
9.3 Analysis of the primary and key secondary variables 24
9.3.1 Variables 24
9.3.2 Statistical model and method of analysis 24
10 Ethical considerations 25
10.1 Regulatory and ethical compliance 25
10.2 Informed consent procedures 25
10.3 Responsibilities of the investigator and IRB/IEC 25
10.4 Publication of study protocol and results 25
11 Protocol adherence 26
11.1 Protocol amendments 26
12 Reference 26

Protocol synopsis

Full title	Early nutritional intervention in patients with locally advanced non-small cell lung cancer receiving concurrent chemoradiotherapy: a single-arm phase II prospective study
Background	Nutritional deficits pose a substantial risk, ranging from 45% to 65%, in patients with lung cancer, significantly affecting their quality of life, anticancer treatment efficacy, and long-term survival. Previous studies suggest that oral nutritional supplements (ONS) can enhance nutritional and protein intake in lung cancer patients. However, the impact of ONS on patient functionality, treatment efficacy, and long-term survival remains uncertain. This study aims to elucidate the comprehensive effects of ONS in the context of lung cancer, shedding light on its potential implications for patient care and outcomes.<br>This prospective, single-arm phase II study aims to investigate the potential benefits of early nutritional intervention in locally advanced non-small cell lung cancer (LANSCLC) patients undergoing definitive concurrent chemoradiotherapy (CCRT). The research seeks to assess whether early nutritional intervention can improve patient outcomes in terms of weight management, nutritional status, quality of life, short-term treatment response, and long-term survival.
Objectives	This study is designed as a single-arm clinical trial to evaluate the efficacy of nutritional intervention in LANSCLC patients undergoing CCRT. In addition to evaluating the prospective study cohort, a historical control group will be incorporated into the analysis plan as part of a pre-specified comparison. The historical control group will consist of patients who had undergone similar CCRT regimens in prior institutional studies but did not receive structured nutritional interventions.<br>Primary endpoint: incidence of weight loss≥5% during the CCRT.<br>Secondary endpoints: nutritional status indicators: hemoglobin levels, serum albumin levels, changes in nutritional parameters, and patient-generated subjective global assessment (PG-SGA) scores. Quality of Life (QoL) scores. Treatment-related adverse effects.<br>Exploratory analysis: gut microbiota analysis.
Study type	Interventional
Study design	Prospective, single-arm Phase II Study
Study Population	LANSCLC patients undergoing definitive CCRT
Sample Population	We hypothesized that the prevalence of weight loss ≥5% at the end of the treatment could be reduced from the 22% reported in previous studies to 10% in the current study. To achieve 80% power for detecting this expected improvement at a one-tailed 0.05 significance level, we needed to enroll 61 patients. Accounting for a 10% dropout rate, the planned enrollment required 67 patients.
Key inclusion criteria	• Age range: 18-75 years.<br>• Patients with LANSCLC (stage IIIA-IIIC) undergoing CCRT.<br>• Pre-treatment PG-SGA grade A-B.<br>• ECOG performance status score of 0-1.<br>• Expected survival of at least six months.<br>• Absence of contraindications for definitive CCRT.

Key exclusion criteria	• Severe impairment of intestinal function or inability to tolerate enteral nutrition.<br>• Severe vomiting, gastrointestinal bleeding, or intestinal obstruction.<br>• Patients with extremely severe malnutrition unable to tolerate CCRT.
Study treatment	• Radiotherapy: hypofractionated radiotherapy with a hypofractionated boost using intensity-modulated radiotherapy techniques. Patients will receive a total dose of 66-68 Gy administered in 17-22 daily fractions to planning target volume.<br>• Concurrent chemotherapy: weekly administrations of docetaxel (25 mg/m^2) and nedaplatin (25 mg/m^2) during radiation therapy<br>• Nutritional intervention: individualized nutrition counseling and ONS from the initiation of CCRT to 2 weeks after its completion. Weekly counseling sessions will be conducted by both doctors and nurses, aim to educate patients on regulating their regular dietary intake. Patients will receive ONS enriched with branched-chain amino acids.
Key evaluation criteria	• Nutritional Indicators: Weight, hemoglobin levels, serum albumin levels, other nutritional parameters, PG-SGA.<br>• QoL scores.<br>• Assessment of antitumor efficacy: overall survival (OS) and progression-free survival (PFS).<br>• Safety evaluation: National Cancer Institute-Common Terminology Criteria for Adverse Events (NCI-CTCAE) version 5.0.
Statistics	Descriptive statistics are applied for the analysis of the baseline characteristics and treatment-related toxicities. Median values are compared using the Wilcoxon test, and proportions are compared using the Chi-square test. Survival rates are estimated using the Kaplan-Meier method and differences in survival curves are compared through the log-rank test. A p-value <0.05 is considered statistically significant

1 Introduction

Lung cancer ranks among the most prevalent malignancies globally, with 1.8 million new cases reported in 2012, constituting 12.9% of all newly diagnosed malignant tumors. Thoracic radiotherapy serves as a curative treatment for unresectable stage III non-small cell lung cancer (NSCLC), inoperable stage I NSCLC, and limited-stage small cell lung cancer. Acute toxicities associated with thoracic radiotherapy include nausea, fatigue, and radiation esophagitis, which may impact patient nutrition, leading to weight loss and malnutrition[1]. Previous studies have indicated that the risk of malnutrition in lung cancer patients is as high as 45-65%. One-third of lung cancer patients experience malnutrition before radiotherapy, while another one-third undergo significant weight loss during the radiotherapy process[2, 3].

Malnutrition significantly impacts the prognosis of lung cancer patients. Luo et al.[4] conducted a retrospective analysis of 110 newly diagnosed non-small cell lung cancer (NSCLC) patients, employing survival analysis and multivariate analysis to examine the relationship between blood cell counts, nutritional status, tumor staging, and prognosis. They identified tumor staging and nutritional status as independent predictors of NSCLC survival. Similarly, Sánchez-Lara et al.[5], in evaluating nutrition-related parameters such as C-reactive protein, blood cell counts, serum albumin, IL-6, and TNF-α levels, confirmed malnutrition as an independent prognostic factor for advanced-stage NSCLC patients. They suggested prospective research to explore the impact of different nutritional therapies on prognosis. Our previous study also indicates that lung cancer patients undergoing definitive intensity-modulated radiotherapy (IMRT) and concurrent chemotherapy face a higher risk of developing radiation pneumonitis (RP) if they are malnourished[6].

In patients with head and neck as well as gastrointestinal malignancies, individualized nutritional counseling and interventions have been shown to improve nutritional status and prognosis[7-9]. Oral nutritional supplements (ONS) are the preferred nutritional supplementation method for patients with normal gastrointestinal function. However, in lung cancer patients, particularly those undergoing radiotherapy, there is limited available data. Studies indicate that nutritional counseling and/or ONS can enhance nutritional and protein intake in lung cancer patients. Yet, the impact on patient functionality, anti-tumor efficacy, and survival outcomes remains unclear[10]. Additionally, there is no consensus on the specific timing and

intervention goals for nutritional support. Lung cancer patients undergoing concurrent chemoradiotherapy typically experience grade 2 or higher radiation-induced esophagitis around 2 weeks into treatment, leading to swallowing difficulties, reduced voluntary food intake, and weight loss. This symptomatology usually persists for 2-3 weeks after the completion of radiotherapy. Clinically, healthcare providers often initiate oral nutritional supplementation when patients exhibit grade 2 esophagitis. However, existing data suggests that weight loss may occur in lung cancer patients before the onset of radiation-induced esophagitis, and early weight loss is identified as one of the adverse prognostic factors[11]. This underscores the necessity for early nutritional intervention.

Building upon the aforementioned issues, this study aims to investigate the impact of early nutritional intervention on LANSCLC patients undergoing CCRT. Specifically, we aim to explore whether early nutritional intervention can improve: (1) weight management: assessing the effect of early nutritional intervention on preventing or mitigating weight loss in patients. (2) nutritional status: evaluating the influence of early nutritional intervention on maintaining or enhancing the nutritional status of the patients. (3) quality of life: investigating whether early nutritional intervention contributes to improvements in the overall quality of life for the patients. (4) short-term treatment response: examining the potential positive effects of early nutritional intervention on the immediate therapeutic outcomes of CCRT. (5) long-term survival: assessing whether early nutritional intervention has a lasting impact on the long-term survival outcomes of the patients. Through comprehensive monitoring and analysis, this research aims to provide valuable insights into the potential benefits of early nutritional intervention in the context of definitive CCRT for LANSCLC patients.

2 Study objectives

This phase II study aimed to evaluate the impact of early nutritional intervention on the nutritional status and survival of LANSCLC patients undergoing definitive CCRT.

2.1 Primary endpoint

- incidence of weight loss ≥ 5% during the CCRT.

2.2 Secondary endpoints

- nutritional status indicators: hemoglobin levels, serum albumin levels, changes in nutritional parameters, and patient-generated subjective global assessment (PG-SGA)

scores.

- quality of life (QoL) scores.
- treatment-related adverse effects.
- survival outcomes.

2.3 Exploratory analysis

- gut microbiota analysis.

3 Investigational plans

3.1 Study design

This single-arm phase II prospective study is to determine the efficacy of early nutritional intervention during concurrent chemoradiotherapy for local advanced non-small cell lung cancer. Patients in the study group received early nutritional intervention, including individualized nutrition counseling and oral nutritional supplements from the initiation of CCRT to 2 weeks after its completion. Weekly counseling sessions, conducted by both doctors and nurses, aimed to educate patients on regulating their regular dietary intake to meet specific energy, protein, and other macronutrient requirements. Dietary advice provided precise instructions on food type and quantity, meal frequency, and calorie or protein intake to ensure a daily energy intake of approximately 30 kcal/kg. All patients received definitive thoracic radiotherapy with total radiation doses of 60-68 Gy, concurrent with weekly docetaxel (25mg/m^2) and nedaplatin (25mg/m^2).

3.2 Research progress and completion time

The study is expected to be 2 years of enrollment, with all patients followed up for 3 years after completion of treatment.

3.3 Comparison with historical control group

This study is designed as a single-arm clinical trial to evaluate the efficacy of nutritional intervention in patients undergoing CCRT. In addition to evaluating the prospective study cohort, a historical control group will be incorporated into the analysis plan as part of a pre-specified comparison. The historical control group will consist of patients who had undergone similar CCRT regimens in prior institutional studies or clinical practice but did not receive structured nutritional interventions.

3.3.1 Purpose of the historical control group

The primary purpose of including a historical control group is to provide context for the outcomes observed in the study group, particularly in terms of weight loss and nutritional outcomes. This comparison is intended to highlight the potential benefits of the intervention relative to standard care practices historically implemented in similar populations.

3.3.2 Selection of historical control group

Eligibility Criteria: Patients in the historical control group will be selected based on the same inclusion and exclusion criteria applied to the prospective study cohort, ensuring comparable baseline characteristics such as age, sex, disease stage, and ECOG performance status.

Data Source: The historical control group data will be sourced from previously conducted prospective studies. Data quality and completeness will be reviewed to ensure reliability and validity for comparative analysis.

Matching and Adjustment: Baseline characteristics between the study and historical control groups will be assessed. Propensity score matching will be planned to adjust for potential confounding factors. Propensity scores will be calculated using a logistic regression model that integrated the following variables: age, gender, ECOG PS, smoking history, diabetes history, clinical Tumor-Node-Metastasis staging, histologic subtypes, and PG-SGA.

3.3.3 Presentation of Results

Results from both the study group and the historical control group will be presented side-by-side to allow direct comparison. Statistical comparisons will be performed to evaluate the differences between groups.

3.3.4 Interpretation of comparative results

The results from the prospective study cohort will be presented first, followed by comparative analyses with the historical control group. While direct comparisons between the groups will provide valuable insights into the potential benefits of the intervention, the non-randomized nature of the historical control group will be clearly acknowledged. These analyses aim to identify trends and generate hypotheses for future randomized controlled trials, rather than establish definitive causal relationships.

4 Population

4.1 Sample size

We hypothesized that the prevalence of weight loss ≥5% at the end of the treatment could be reduced from the 22% reported in previous studies to 10% in the current study. To achieve 80% power for detecting this expected improvement at a one-tailed 0.05 significance level, we needed to enroll 61 patients. Accounting for a 10% dropout rate, the planned enrollment required 67 patients.

4.2 Inclusion criteria

Patients eligible for inclusion in this study have to fulfill **all** of the following criteria:

1. Age range: 18-75 years.
2. Patients with LANSCLC (stage IIIA-IIIC) undergoing CCRT.
3. Pre-treatment PG-SGA grade A-B.
4. ECOG performance status score of 0-1.
5. Expected survival of at least six months.
6. Absence of contraindications for definitive CCRT.

4.3 Exclusion criteria

Patients fulfilling **any** of the following criteria are not eligible for inclusion in this study.

1. Severe impairment of intestinal function or inability to tolerate enteral nutrition.
2. Severe vomiting, gastrointestinal bleeding, or intestinal obstruction.
3. Patients with extremely severe malnutrition unable to tolerate CCRT.

4.4 Premature patient withdrawal

Patients may voluntarily withdraw from the study for any reason at any time. They may be considered withdrawn if they state an intention to withdraw, fail to return for visits, or become lost to follow-up for any other reason.

If premature withdrawal occurs for any reason, the investigator must make every effort to determine the primary reason for a patient’s premature withdrawal from the study and record this information on the Study Completion Case Report/Record Form (CRF).

The investigator withdraws the subject from study under the following circumstances:

- Withdrawal of informed consent.
- Treatment cannot be performed as required by the study protocol.
- Disease progress during treatment.
- The patient is pregnant or not using adequate contraception.
- Any other protocol deviation that results in a significant risk to the patient's safety

For patients who are lost to follow-up (i.e. those patients whose status is unclear because they fail to appear for study visits without stating an intention to withdraw), the investigator should show "due diligence" by documenting in the source documents steps taken to contact the patient, e.g. dates of telephone calls, registered letters, etc.

5 Treatment

5.1 Nutrition intervention

Patients in the study group will receive early nutritional intervention, including individualized nutrition counseling and oral nutritional supplements from the initiation of CCRT to 2 weeks after its completion. Weekly counseling sessions will last approximately 30 minutes and is conducted by both doctors and nurses. The weekly counseling sessions are separate from the standard weekly on-treatment monitoring appointments. The counseling sessions focus exclusively on nutritional education, personalized advice, and support, while the status checks address overall treatment progress and symptom management. Dietary advices provide precise instructions on food type and quantity, meal frequency, and calorie or protein intake to ensure a daily energy intake of approximately 30 kcal/kg. The nutritional intervention is further standardized to include a protein intake target of 1.2–1.5 g/kg/day to support muscle mass maintenance and recovery. Patients in the study group will receive oral nutritional supplements enriched with branched-chain amino acids. Nutritional supplementation is reviewed and will tailor weekly during the counseling sessions, based on patients' updated nutritional assessments, weight trends, and dietary intake. To ensure adherence, the research team will assess patients' actual energy and nutrient intake weekly through food diaries and nutritional intake records and adjusted dietary recommendations accordingly. In contrast, patients in the historical control group received standard care without specific dietary guidelines or nutrition counseling sessions. However, patients experiencing significant weight loss (≥5%) were managed according to standard of care practices, which included additional dietary

recommendations or support measures. While the control group did not participate in structured nutritional guidance or personalized supplementation plans, they were seen weekly by doctors to address standard care symptoms during treatment.

5.2 Radiotherapy

5.2.1 Postural immobilization

1. During positioning, a position-fixing device must be employed to ensure the repeatability of radiotherapy positioning and treatment accuracy. The specific device may vary across different tumor centers, but the device used during positioning must be consistent with the treatment position. Treatment planning graphics acquisition and delineation of target areas, including Gross Target Volume (GTV), Clinical Tumor Volume (CTV), Planning Target Volume (PTV), and normal organs, will be conducted using simulation positioning CT. During simulation positioning CT scanning, patients should maintain a calm normal breathing state, with the upper boundary at the top of the thyroid and the lower boundary at the bottom of L2, covering the entire chest, with a slice thickness of 3 mm.

2. Enhanced chest CT can be used to guide the delineation of major vessels and mediastinal lymph node regions. In this study, enhanced CT can be utilized for simulation positioning to facilitate target area delineation. If the high electron density of enhanced tissue images affects treatment planning, it can be fused with plain CT for treatment planning.

3. This study encourages the use of FDG-PET/CT for simulation positioning. If this method is employed, the same position-fixing device as in the treatment position must be used. If the scanning position is consistent with the treatment position, the CT component can be used for treatment planning. The study requires the use of 4D-CT scanning and the application of 4D-CT for treatment planning.

5.2.2 Definition of target area

The definition of radiotherapy target area follows ICRU reports 50 and 62.

1. GTV: GTV encompasses all visible tumors, including primary tumors and mediastinal lymph node regions revealed by CT, PET scans, bronchoscopy, or mediastinoscopy. Unless ruled out by cytology, pathology, or PET scans for metastasis, all lymph nodes in the mediastinum with a short axis ≥ 1.0 cm should be included in the GTV. In the presence of

atelectasis in a lung lobe or segment, PET or MR images can be utilized to distinguish atelectasis from tumor involvement.

2. CTV: CTV includes an expansion of 5-10 mm beyond the IGTV and the involved lymph node drainage area, aiming to encompass the microscopic tumor invasion range. In this study, all patients will receive involved-field lymph node drainage area irradiation, without preventive irradiation of the mediastinal and supraclavicular lymph nodes. For N2 patients, even if no abnormalities are observed on imaging, the CTV should also include the ipsilateral hilum adjacent to the tumor. If imaging shows no involvement of the hilum, the lower pole of the ipsilateral hilum is excluded if the primary tumor is located in the upper lobe, and vice versa.

3. PTV: Internal GTV (IGTV) is directly delineated based on the maximum intensity projection images and then fine-tuned using CT scans from all respiratory phases. To establish planning target volume (PTV), a uniform expansion of 5 mm around the IGTV is applied.

4. It is recommended that image-guided technology (KV-X-ray online guidance, Cone-beam CT online or offline guidance) should be to quantify, correct and record the positioning errors.

5. The delineation of the target area and normal tissue should be carried out by the radiotherapy physicians, and it is recommended to improve the accuracy of the delineation of the target area through the consultation of the radiologist.

5.2.3 Radiotherapy plan

In the study, intensity-modulated radiation therapy (IMRT) technology is applied to formulate a radiotherapy plan.

5.2.4 Dosage prescription

All enrolled participants in this study will undergo hypofractionated radiotherapy with a hypofractionated boost using intensity-modulated radiotherapy techniques. The treatment regimen comprises intensity-modulated radiotherapy to the chest, administered once daily for five days a week, with a prescribed dose ranging from 4000 cGy to 5100 cGy over 10-17 fractions. After the first course of radiotherapy, a chest and upper abdominal CT scan will be conducted later. Patients without local progression or distant metastasis will undergo a repeat CT simulation for precise localization and treatment planning before receiving the boost

radiotherapy, with a dose ranging from 1500 cGy to 2800 cGy over 5-7 fractions. The radiotherapy plans satisfied that the prescribed dose covered the 99% volume of GTV and 95% volume of PTV, and the maximal dose is required to be less than 110% of the prescribed dose. The dose calculation should take into account the difference the difference in tissue density in the irradiated area (ie, air density of lung and bone) to correct for tissue heterogeneity.

The maximum dose point and the minimum dose point with the PTV should recorded. The dose distribution inside and outside the PTV must be evaluated, and dose deviations distinguished and adjusted accordingly.

- No deviation: No deviation: ≥99% of the PTV volume receives at least 95% of the prescribed dose. No region within the PTV exceeds 110% of the prescribed dose, and no area $1cm^3$ or larger outside the PTV receives more than 110% of the prescribed dose.
- Slight deviation: Slight deviation: This level of deviation is acceptable but should be minimized. Between 95% and 99% of the PTV volume receives at least 95% of the prescribed dose. An area within the PTV with a volume greater than $1cm^3$ receives more than 110% but less than or equal to 115% of the prescribed dose, and an adjacent area outside the PTV with a volume of $1cm^3$ or less receives more than 110% but less than or equal to 115% of the prescribed dose.
- Serious deviation: This level of deviation is unacceptable. Less than 95% of the PTV volume receives at least 95% of the prescribed dose. An area within the PTV with a volume greater than $1cm^3$ receives more than 115% of the prescribed dose, and an adjacent area outside the PTV with a volume of $1cm^3$ or more receives more than 115% of the prescribed dose.

5.2.5 Organ at risk

When formulating the treatment plan, due consideration should be given to the tolerance of normal tissues. In cases where it is challenging to balance target volume coverage with sparing critical organs, priority should be given to limiting the dose to critical organs. The prioritization order of critical organs in the treatment plan is as follows: 1=spinal cord, 2=bilateral lungs, 3=heart, 4=esophagus, 5=liver.

For spinal cord, the external contour of the vertebral canal should be delineated to represent it.

The maximum allowable dose for the spinal cord is 46 Gy, with a dose exceeding 50 Gy considered a serious deviation.

Concerning the lungs, the volume of both lungs (excluding the planned target area) reaching 20 Gy (V20) should not exceed 35%. V5 should not exceed 70%, and the Mean Lung Dose (MLD) should be kept below 19 Gy.

For the esophagus, the maximum dose should be less than 64 Gy, and V55 (the percentage of the esophagus receiving 55 Gy) should be less than 15%.

The heart needs to be delineated at all levels. The upper boundary should include the funnel part of the right ventricle and the tops of the two atria, making efforts to exclude large blood vessels. The lower boundary, representing the lowest point of the left ventricle, should be separated from the liver. Recommended dose constraints include V40 (the percentage of the heart receiving 40 Gy) being kept below 50%.

As for the liver, the volume of liver receiving 30 Gy (V30) should not exceed 40%.

5.2.6 Delivery of treatment

X-ray with energy ≥6 MV is used for treatment. In the study, we recommend the use of a dynamic multi-leaf linear accelerator, avoiding the use of cyberknife, gamma knife and Tomotherapy. Try to ensure that the enrolled patients receive radiotherapy by the same treatment planning system and the same linear accelerator, and try to complete treatment in the same linear accelerator for each patient (except for forcing majeure external reasons such as long-term mechanical failure). Daily cone-beam CT image guidance is adopted to verify the tumor position and ensure the precision of radiotherapy.

5.2.7 Quality control

Prior to enrolling the first patient, the study center will be evaluated using a simulated case. The radiotherapy plan for the simulated case is reviewed to ensure that the radiotherapy prescription meets protocol requirements. The positioning and radiotherapy plan for each enrolled patient will be reviewed after enrollment.

The schedule quality certification (annual, monthly, weekly, daily) of simulation CT, treatment planning system and linear accelerator will be reviewed before enrollment.

It is required that the electronic documentation of the radiotherapy plan for each enrolled patient must be backed up and archived. Dose-volume histogram (DVH) should describe the target area, the lungs, the lungs, the spinal cord, and the heart. The following dose values should be recorded: the dose at the point of prescription, the minimum dose, the maximum dose and the average dose of the PTV, the maximum dose of the spinal cord, the V20 (volume receiving 20 Gy) and V5 (volume receiving 5 Gy) for both lungs, the mean dose (average dose) and maximum dose for the heart, as well as the maximum dose and mean dose for the esophagus.

5.3 Concurrent chemotherapy

Concurrent chemotherapy involved weekly administrations of docetaxel (25 mg/m^2) and nedaplatin (25 mg/m^2) during radiation therapy. Docetaxel is administered as an infusion after dilution with 0.9% sodium chloride injection, with a final concentration not exceeding 0.9 mg/ml. The infusion should be completed within 1 hour. For cisplatin infusion: cisplatin is diluted in 500 ml of 0.9% sodium chloride injection and administered over a period of 3 hours. The infusion is conducted within a 3-hour timeframe.

6 Adverse events and treatment plan adjustment

All treatment-related acute and late toxicities are graded in terms of the Common Terminology Criteria for Adverse Events (CTCAE) of the National Cancer Institute (version 5.0) and record on the Study Completion CRF. Serious adverse events (SAE) must be reported to the institutional review board within 24h and dealt with properly.

6.1 Definition

6.1.1 Adverse events (AE)

1. An adverse event (AE) is any untoward medical occurrence (i.e., any unfavorable and unintended sign [including abnormal laboratory findings], symptom or disease) in a subject or clinical investigation subject *after providing written informed consent* for participation in the study.

2. The occurrence of adverse events should be sought by non-directive questioning of the patient at each visit during the study. Adverse events also may be detected when they are

volunteered by the patient during or between visits or through physical examination, laboratory test, or other assessments.

3. Abnormal laboratory values or test results constitute adverse events only if they fulfill at least one of the following criteria:

- they induce clinical signs or symptoms
- they are considered clinically significant
- they require therapy

4. Clinically significant abnormal laboratory values or test results should be identified through a review of values outside of normal ranges/clinically notable ranges, significant changes from baseline or the previous visit, or values which are considered to be non-typical in patient with underlying disease. Investigators have the responsibility for managing the safety of individual patient and identifying adverse events.

5. Adverse events should be recorded in the Adverse Events CRF under the signs, symptoms or diagnosis associated with them accompanied by the following information

- the severity grades
- its relationship to the previous study treatment
- its duration (start and end dates) or if the event is ongoing an outcome of not recovered/not resolved should be reported.
- whether it constitutes a SAE
- whether other medication or therapies have been taken (concomitant medication/non-drug therapy)
- its outcome (not recovered/not resolved; recovered/resolved; recovering/resolving, recovered/resolved with sequelae; fatal; or unknown)

6.1.2 Serious adverse events (SAE)

An SAE is any adverse event (appearance of (or worsening of any pre-existing) undesirable sign(s), symptom(s) or medical conditions(s) which meets any one of the following criteria:

- is fatal or life-threatening.
- results in persistent or significant disability/incapacity.
- constitutes a congenital anomaly/birth defect

- requires inpatient hospitalization or prolongation of existing hospitalization
- is medically significant, i.e. defined as an event that jeopardizes the patient or may require medical or surgical intervention to prevent one of the outcomes listed above.

6.2 Toxicity of radiotherapy and management of radiation-related side effects

1. Unplanned interruptions in radiation therapy should be avoided whenever possible.

2. Hematologic toxicity: In the event of febrile neutropenia, confirmed infection, or bleeding, radiation therapy should be interrupted until toxicity recovers to Grade 2 or below (including Grade 2). The duration of radiation therapy interruption should be ≤14 days. If radiation therapy interruption is caused by treatment-related adverse reactions, drug treatment should also be correspondingly suspended. Efforts should be made to minimize radiation therapy interruptions. To ensure the smooth implementation of radiation therapy, preventive measures and nutritional support therapy can be employed. In rare cases, such as when patients experience treatment-unrelated or unforeseen adverse reactions, the researcher may judiciously consider whether treatment interruption is necessary. Treatment can resume after adverse reactions have recovered to Grade 2 or below. The maximum duration of radiation therapy interruption should not exceed 2 weeks.

3. For the management of radiation pneumonitis and esophagitis, refer to the treatment methods in the following table:

	NCI CTCAE 5.0	Management
Radiation esophagitis	Grade 1: No symptoms, only visible through pathology, imaging, or endoscopy.	No specific intervention is recommended.
	Grade 2: Symptoms present, such as changes in diet or swallowing. Oral supplementation may be sufficient; intravenous fluids needed for less than 24 hours.	Symptomatic relief measures are initiated.
	Grade 3: Symptoms are severe, with significant changes in diet or swallowing. Inadequate oral intake of	Topical treatment with lidocaine gel plus calcium carbonate or antacids, along with sucralfate. Consideration of stopping radiation therapy and/or

	calories or fluids, requiring intravenous fluids. Enteral nutrition via a gastric tube or parenteral nutrition for more than 24 hours may be necessary.	medication until symptoms reach Grade 2 or below.
	Grade 4: Life-threatening, with conditions such as obstruction or perforation.	Use of ranitidine or other H2 receptor blockers, or proton pump inhibitors. Consideration of stopping radiation therapy and/or medication until symptoms reach Grade 2 or below.
Radiation pneumonitis	Grade 1: No symptoms, only visible through imaging.	Emphasize the importance of rest, infection prevention, and strongly recommend that patients who smoke cease smoking during the course of radiation therapy.
	Grade 2: Symptoms present, with changes in respiratory function but not affecting daily life.	Symptomatic management includes oxygen therapy and nutritional support.
	Grade 3: Symptoms are present and impact daily life as respiratory function changes. Oxygen supplementation is needed.	Antimicrobial therapy: Promptly identify the causative pathogen and select antibiotics based on sensitivity testing when infection occurs. Consider discontinuing radiation therapy and/or medication.
	Grade 4: Life-threatening symptoms requiring ventilatory support.	Corticosteroids. Consider discontinuing radiation therapy.

To prevent symptomatic radiation-induced lung injury, it is essential to maximize the protection of normal lung tissue, especially in patients with pre-existing poor lung function, during the development of the radiation therapy plan. Throughout the treatment, monitor changes in patient symptoms and signs (respiratory sounds, rales) and conduct timely blood analyses, chest X-rays or CT scans, blood gas analyses, and other tests to comprehensively assess the patient's lung function. High-risk patients should be vigilant for acute pulmonary embolism.

6.3 Toxicity of chemotherapy and chemotherapy dose adjustment

1. In monotherapy with docetaxel, dose-limiting toxicity is neutropenia. Other possible adverse events include allergies, skin reactions, gastrointestinal toxicity (nausea, vomiting, oral mucositis, diarrhea), hair loss, muscle weakness, mild injection site reactions (venous

inflammation), peripheral neuropathy, and fluid retention/edema.

2. In monotherapy with cisplatin, major adverse events include ototoxicity, peripheral neuropathy, renal failure, and vomiting. If a patient experiences multiple toxicities, and the dose adjustment principles for each toxicity differ, choose the minimum dose. If the dose of the investigational drug is reduced due to drug-related toxicity, the dose cannot be increased even after the toxicity disappears.

3. All patients receive the maximum chemotherapy dose, and if necessary, the dose is adjusted based on the most severe hematologic or other toxicities. If a patient needs a dose reduction, they will continue to receive the reduced dose in subsequent treatment cycles. If a patient experiences multiple toxicities, and the dose adjustment principles for each toxicity differ, choose the minimum dose. If a patient has already undergone two dose reductions (for the same drug), chemotherapy must be discontinued if a third dose reduction is required due to toxicity reactions. Chemotherapy can be delayed for up to two weeks at most; otherwise, it must be discontinued.

7 Visit schedule and assessments

7.1 The baseline assessment

All patients must complete the following required tests before being considered for inclusion in the study. The baseline assessment is generally required to be completed with 21 days before treatment.

Subjects		Time
Informed consent		Prior to treatment
Medical history & physical examination	Medical history: history of concomitant disease and medication.<br>Physical examination: height, weight, Body Mass Index (BMI), ECOG score, symptom, and systemic examination	Within 7 days prior to treatment
Blood routine	Hemoglobin, Platelets, White Blood Cells, Neutrophils.	Within 7 days prior to treatment
Serological tests	Nutritional markers (prealbumin, transferrin, retinol-binding protein), C-reactive protein, liver and kidney function, lipid profile, blood glucose, electrolytes.	Within 7 days prior to treatment
	a. A CT examination of the chest and upper abdomen is conducted, with a routine enhanced	Within 21 days prior to treatment

Imaging examinations	scan following the plain scan<br>b. Lower abdominal and pelvic CT scan (if necessary)<br>c. Bone scan, optional if necessary to exclude bone metastases<br>d. Any other necessary examinations	
Electrocardiogram	12-lead electrocardiogram	Within 7 days prior to treatment
Quality of life (QOL)	The QOL is evaluated using the European Organization for Research and Treatment of Cancer QOL questionnaire version 3.0 (QLQ-C30)	Within 7 days prior to treatment
Nutritional risk assessment	PG-SGA, Nutritional Risk Screening 2002 (NRS-2002)	Within 7 days prior to treatment
Other examinations based on clinical need		

7.2 The assessment during the treatment

Subjects		Time
Medical history & physical examination	Medical history: concomitant medication.<br>Physical examination: height, weight, BMI, ECOG score, and systemic examination	Once a week, at the end of CCRT
Blood routine	Hemoglobin, Platelets, White Blood Cells, Neutrophils	Once a week, at the end of CCRT
Serological tests	Nutritional markers (prealbumin, transferrin, retinol-binding protein), C-reactive protein, liver and kidney function, lipid profile, blood glucose, electrolytes.	Once a week, at the end of CCRT
Electrocardiogram	12-lead electrocardiogram	Once a week, at the end of CCRT
Nutritional risk assessment	PG-SGA, NRS-2002	Once every two weeks, at the end of CCRT
Quality of life (QOL)	The QOL is evaluated using the European Organization for Research and Treatment of Cancer QOL questionnaire version 3.0 (QLQ-C30)	After the completion of treatment
Other examinations based on clinical need		
Assessment of toxicity		Once a week

7.3 The assessment after the completion of treatment

Subjects		Time
Medical history & physical	Medical history: concomitant medication.<br>Physical examination: height, weight, BMI,	During each follow-up examination

examination	ECOG score, and systemic examination	
Blood routine	Hemoglobin, Platelets, White Blood Cells, Neutrophils	One month after treatment
Serological tests	Nutritional markers (prealbumin, transferrin, retinol-binding protein), C-reactive protein, liver and kidney function, lipid profile, blood glucose, electrolytes.	One month after treatment
Imaging examinations	a. A CT examination of the chest and upper abdomen is conducted, with a routine enhanced scan following the plain scan<br>b. Lower abdominal and pelvic CT scan (if necessary)<br>c. Bone scan, optional if necessary to exclude bone metastases<br>d. Any other necessary examinations	Every 3 months for the first 2 years, every 6 months for years 3-5, and yearly thereafter
Quality of life (QOL)	The QOL is evaluated using the European Organization for Research and Treatment of Cancer QOL questionnaire version 3.0 (QLQ-C30)	At 6 months after the completion of the treatment
Nutritional risk assessment	PG-SGA, NRS-2002	At 6 months after the completion of the treatment
Survival status assessment	Outpatient or telephone follow-up	Every 3 months for the first 2 years, every 6 months for years 3-5, and yearly thereafter
Assessment of toxicity		During each follow-up examination
Other examinations based on clinical need		

Monitor research-related adverse events and document outcomes until they resolve, are determined to be unrelated, or are determined by the investigator not to require further follow-up from a medical perspective. Any accompanying treatment for these adverse events should be recorded in the CRF.

7.4 Quality of life score

The quality of life of the patients is assessed using the EORTC Quality of Life Measurement Scale QLQ-C30 at enrollment, at baseline, end of the treatment, and 6 months after the completion of treatment.

7.5 Follow up

One month after the completion of CCRT, the treatment response is assessed. All patients are then followed every 3 months for the first 2 years, every 6 months for years 3-5, and yearly thereafter. Follow-up workup included patients' medical history, physical examination, laboratory tests, CT scans of the chest.

8 Evaluation of the research results

8.1 Efficacy

8.1.1 Incidence of weight loss

Incidence of weight loss are calculated.

8.1.2 Nutritional status

Nutritional parameters, such as hemoglobin, lymphocyte, pre-albumin, albumin, and inflammation biomarkers, such as CRP, and NLR. PG-SGA are also evaluated.

8.1.3 Survival outcomes

1. Progression-free survival (PFS): PFS is calculated from the completion of radiotherapy to the date of locoregional failure or distant metastasis or death, whichever occurs first.

2. Overall survival (OS): OS is calculated from the completion of radiotherapy to the date of death from any cause or censored at the last follow-up.

8.2 Safety assessment

8.2.1 Overall safety

With the exception of nausea and vomiting, all toxicities observed within 24 hours of administration should be recorded.

Adverse events/symptoms: Adverse events and symptoms associated with the disease should be regularly assessed according to NCI CTCAE version 5.0.

8.2.2 Laboratory evaluations

The change in hematology, chemistry will be assessed at all visits as an overall measure of safety over time.

9 Statistics

9.1 Analysis sets

1. Intent-to-treat population (ITT): The ITT set consists of all subjects who signed informed consent and are included regardless of whether they completed the entire treatment process. ITT sets are used for safety and efficacy analysis.

2. Per-protocol population (PP): The PP set is defined as a subgroup of ITT set. PP set refers to those who meet the conditions of ITT set and complete all treatment as per protocol. It is generally used for the primary efficacy analysis.

9.2 Patient demographics and other baseline characteristics

Summary statistics will be provided for patient demographics (age, sex, race, ethnicity, height, weight and BMI) and other baseline disease characteristics such as PG-SGA score. Continuous variables will be presented with mean, median, 25th percentile, 75th percentile, standard deviation, minimum and maximum, and the number of non-missing observations. Categorical data will be displayed via absolute and relative frequencies for each category (including a category labeled as ‘missing’ when appropriate).

9.3 Analysis of the primary and key secondary variables

9.3.1 Variables

The primary efficacy variable is incidence of weight loss ≥5% during the CCRT. The key secondary variables are nutritional biomarkers, PG-SGA, QOL scores, toxicities, OS and PFS.

9.3.2 Statistical model and method of analysis

Descriptive statistics are applied for the analysis of the baseline characteristics and treatment-related toxicities. Median values are compared using the Wilcoxon test, and proportions are compared using the Chi-square test. Survival rates are estimated using the Kaplan-Meier method and differences in survival curves are compared through the log-rank test. A p-value <0.05 is considered statistically significant.

10 Ethical considerations

10.1 Regulatory and ethical compliance

This clinical study is designed and shall be implemented and reported in accordance with the International Conference on Harmonization (ICH) Harmonized Tripartite Guidelines for Good Clinical Practice, with applicable local regulations, and with the ethical principles laid down in the Declaration of Helsinki.

10.2 Informed consent procedures

Eligible patients may only be included in the study after providing written (witnessed, where required by law or regulation), IRB/IEC-approved informed consent, or, if incapable of doing so, after such consent has been provided by a legally acceptable representative of the patient. In cases where the patient's representative gives consent, the patient should be informed about the study to the extent possible given his/her understanding. If the patient is capable of doing so, he/she should indicate assent by personally signing and dating the written informed consent document or a separate assent form. Informed consent must be obtained before conducting any study-specific procedures (i.e. all of the procedures described in the protocol). The process of obtaining informed consent should be documented in the patient source documents.

10.3 Responsibilities of the investigator and IRB/IEC

The protocol and the proposed informed consent form must be reviewed and approved by a properly constituted Institutional Review Board/Independent Ethics Committee/Research Ethics Board (IRB/IEC) before study start. Prior to study start, the investigator is required to sign a protocol signature page confirming his/her agreement to conduct the study in accordance with these documents and all.

10.4 Publication of study protocol and results

Upon study completion and finalization of the study report the results of this trial will be either submitted for publication and/or posted in a publicly accessible database of clinical trial results.

11 Protocol adherence

Investigators ascertain they will apply due diligence to avoid protocol deviations.

This protocol defines the study objectives, the study procedures and the data to be collected on study participants. Under no circumstances should an investigator collect additional data or conduct any additional procedures for any research related purpose involving any investigational drugs.

If the investigator feels a protocol deviation would improve the conduct of the study this must be considered a protocol amendment, and unless such an amendment is approved by the IRB/IEC it cannot be implemented. All significant protocol deviations will be recorded and reported in the CSR.

11.1 Protocol amendments

Any change or addition to the protocol can only be made in a written protocol amendment that must be approved by the IRB/IEC. Only amendments that are required for patient safety may be implemented prior to IRB/IEC approval. Notwithstanding the need for approval of formal protocol amendments, the investigator is expected to take any immediate action required for the safety of any patient included in this study, even if this action represents a deviation from the protocol. In such cases, the IRB/IEC at the study site should be informed within 10 working days.

12 Reference

[1] Bovio G, Montagna G, Bariani C, Baiardi P. Upper gastrointestinal symptoms in patients with advanced cancer: relationship to nutritional and performance status. *Supportive care in cancer : official journal of the Multinational Association of Supportive Care in Cancer* 2009;17(10):1317-24. doi:10.1007/s00520-009-0590-x.

[2] Kiss N, Isenring E, Gough K, Krishnasamy M. The prevalence of weight loss during (chemo)radiotherapy treatment for lung cancer and associated patient- and treatment-related factors. *Clinical nutrition (Edinburgh, Scotland)* 2014;33(6):1074-80. doi:10.1016/j.clnu.2013.11.013.

[3] Unsal D, Mentes B, Akmansu M, Uner A, Oguz M, Pak Y. Evaluation of nutritional status in cancer patients receiving radiotherapy: a prospective study. *American journal of clinical oncology* 2006;29(2):183-8. doi:10.1097/01.coc.0000198745.94757.ee.

[4] Luo J, Chen YJ, Narsavage GL, Ducatman A. Predictors of survival in patients with non-small cell lung cancer. *Oncology nursing forum* 2012;39(6):609-16. doi:10.1188/12.Onf.609-616.

[5] Sánchez-Lara K, Turcott JG, Juárez E, et al. Association of nutrition parameters including bioelectrical impedance and systemic inflammatory response with quality of life and prognosis in patients with advanced non-small-cell lung cancer: a prospective study. *Nutrition and cancer* 2012;64(4):526-34.

doi:10.1080/01635581.2012.668744.
[6] Ma L, Ye W, Li Q, et al. Subjective Global Assessment (SGA) Score Could Be a Predictive Factor for Radiation Pneumonitis in Lung Cancer Patients With Normal Pulmonary Function Treated by Intensity-Modulated Radiation Therapy and Concurrent Chemotherapy. *Clinical lung cancer* 2018;19(2):e211-e7. doi:10.1016/j.cllc.2017.09.001.
[7] Isenring EA, Capra S, Bauer JD. Nutrition intervention is beneficial in oncology outpatients receiving radiotherapy to the gastrointestinal or head and neck area. *British journal of cancer* 2004;91(3):447-52. doi:10.1038/sj.bjc.6601962.
[8] Ravasco P, Monteiro-Grillo I, Marques Vidal P, Camilo ME. Impact of nutrition on outcome: a prospective randomized controlled trial in patients with head and neck cancer undergoing radiotherapy. *Head & neck* 2005;27(8):659-68. doi:10.1002/hed.20221.
[9] Ravasco P, Monteiro-Grillo I, Vidal PM, Camilo ME. Dietary counseling improves patient outcomes: a prospective, randomized, controlled trial in colorectal cancer patients undergoing radiotherapy. *Journal of clinical oncology : official journal of the American Society of Clinical Oncology* 2005;23(7):1431-8. doi:10.1200/jco.2005.02.054.
[10] Kiss NK, Krishnasamy M, Isenring EA. The effect of nutrition intervention in lung cancer patients undergoing chemotherapy and/or radiotherapy: a systematic review. *Nutrition and cancer* 2014;66(1):47-56. doi:10.1080/01635581.2014.847966.
[11] Sanders KJ, Hendriks LE, Troost EG, et al. Early Weight Loss during Chemoradiotherapy Has a Detrimental Impact on Outcome in NSCLC. *Journal of thoracic oncology : official publication of the International Association for the Study of Lung Cancer* 2016;11(6):873-9. doi:10.1016/j.jtho.2016.02.013.
